# Shedding Light on the African Enigma: In Vitro Testing of *Homo sapiens-Helicobacter pylori* Coevolution

**DOI:** 10.3390/microorganisms9020240

**Published:** 2021-01-25

**Authors:** Bruno Cavadas, Marina Leite, Nicole Pedro, Ana C. Magalhães, Joana Melo, Marcelo Correia, Valdemar Máximo, Rui Camacho, Nuno A. Fonseca, Ceu Figueiredo, Luísa Pereira

**Affiliations:** 1 i3S—Instituto de Investigação e Inovação em Saúde, Universidade do Porto, 4200-135 Porto, Portugal; mleite@ipatimup.pt (M.L.); npedro@ipatimup.pt (N.P.); acmagalhaes@ipatimup.pt (A.C.M.); jmelo@ipatimup.pt (J.M.); mcorreia@ipatimup.pt (M.C.); vmaximo@ipatimup.pt (V.M.); cfigueiredo@ipatimup.pt (C.F.); luisap@ipatimup.pt (L.P.); 2 IPATIMUP—Instituto de Patologia e Imunologia Molecular, Universidade do Porto, 4200-135 Porto, Portugal; 3 ICBAS—Instituto de Ciências Biomédicas Abel Salazar, Universidade do Porto, 4050-313 Porto, Portugal; 4 FMUP—Faculdade de Medicina, Universidade do Porto, 4200-319 Porto, Portugal; 5 FEUP—Faculdade de Engenharia, Universidade do Porto, 4200-465 Porto, Portugal; rcamacho@fe.up.pt; 6 INESC TEC—Instituto de Engenharia de Sistemas e Computadores, Tecnologia e Ciência, Universidade do Porto, 4200-465 Porto, Portugal; 7 CIBIO—Centro de Investigação em Biodiversidade e Recursos Genético, Universidade do Porto, 4485-661 Vairão, Portugal; nuno.fonseca@cibio.up.pt

**Keywords:** coevolution, *Helicobacter pylori*, *Homo sapiens*, genome-wide gene expression, ancestry background, innate immune response

## Abstract

The continuous characterization of genome-wide diversity in population and case–cohort samples, allied to the development of new algorithms, are shedding light on host ancestry impact and selection events on various infectious diseases. Especially interesting are the long-standing associations between humans and certain bacteria, such as the case of *Helicobacter pylori*, which could have been strong drivers of adaptation leading to coevolution. Some evidence on admixed gastric cancer cohorts have been suggested as supporting *Homo*-*Helicobacter* coevolution, but reliable experimental data that control both the bacterium and the host ancestries are lacking. Here, we conducted the first in vitro coinfection assays with dual human- and bacterium-matched and -mismatched ancestries, in African and European backgrounds, to evaluate the genome wide gene expression host response to *H. pylori*. Our results showed that: (1) the host response to *H. pylori* infection was greatly shaped by the human ancestry, with variability on innate immune system and metabolism; (2) African human ancestry showed signs of coevolution with *H. pylori* while European ancestry appeared to be maladapted; and (3) mismatched ancestry did not seem to be an important differentiator of gene expression at the initial stages of infection as assayed here.

## 1. Introduction

Coevolution is a biological term coined in 1964 by Ehrlich and Raven [1] to describe relationships between two entities where selective pressures are exerted on each other’s evolution. Coevolution occurs in many forms of mutualism, host–parasite, and predator-prey relationships, as well as competition within or between species. The most extreme examples of exquisite adaptation, displaying evidence of tightly coevolved morphology, physiology, and behavior, are the symbiotic integrations of mitochondria and chloroplasts in eukaryotic cells [2]. Improvements in mathematical models [3] have been showing that the expected stable adaptive peaks attainable through coevolution are rarely maintained, as the selective landscapes are under continual change through reciprocal selection on the species themselves. Additionally, further shaping is modeled by the geographic structure of the species, with various selection mosaics evolving via different evolutionary trajectories, which will be further changed by the action of gene flow and random genetic drift [3]. Therefore, coevolving species are moderately maladapted most of the time.

The rational of adaptation and maladaptation is beginning to be introduced into the field of infectious diseases caused by long term host–parasite relationships and should be explored in explaining disease emergence [4]. Kodaman et al. [5] also advocate that genome-by-genome interactions should be incorporated into genetic models of disease caused by infectious agents, such as *Helicobacter pylori*, *Mycobacterium tuberculosis* and human papillomavirus. These pathogens have likely been infecting the human species since before/after its origins in the African continent, and definitely prior to the out-of-Africa migration that led to the population structure of humans and its parasites: 88,000–116,000 years ago for *H. pylori* [6]; 70,000 years ago for *M. tuberculosis* [7]; and more than 200,000 years ago for HPV16 and HPV18 [8]. The longer coexistence in Africa leads to expectations of better adaptation in this continent, while the extreme bottleneck of the out-of-Africa migration, and the selective pressure of the new European and Asian environments, generated maladaptations that had/have to evolve to new adaptive situations [9]. Later on, migrations between the structured population groups propitiated the encounter of mismatched human and pathogen ancestries or situations of disruptive coevolution.

Of those three long-term human pathogens, *H. pylori* has displayed, so far, the best epidemiological evidence in favor of coevolution. This type of bacteria chronically infects almost 50% of the worldwide population [10], causing superficial gastritis in all infected individuals. While the great majority of infected individuals do not develop severe clinical outcomes, about 10–20% will develop peptic ulcer disease, and ~1% can develop gastric carcinoma [11]. Opposing these disease-causing effects, it is possible that *H. pylori* confers fitness benefits to its host by being protective against gastroesophageal diseases [12,13,14], as well as against asthma and other allergies [15]. Virulence factors in *H. pylori* (specially the presence of the CagA oncoprotein and of the toxic form of the vacuolating cytotoxin VacA) and host polymorphisms in genes that alter cytokine expression (IL-1β, TNF-α, IL-10, IL-8, and COX-2) have been linked to an increased risk for gastric cancer development [16]. However, studies on these factors in isolation generally fail to explain the difference in susceptibilities to gastric cancer seen between individuals and ethnicities.

Phylogeographic analysis confirmed the strong concordance between *H. pylori* clusters and the geographical locations from where the human samples had been collected [17,18]: *hp*Europe, isolated from Europeans, Middle Easterns and Indians; *hp*Africa1 from Morocco, Senegal, Burkina Faso, and South Africa; *hp*NEAfrica, in Ethiopia, Somalia, Sudan and Nigerians; *hp*Asia2, Northern India, Bangladesh, Thailand, and the Philippines; and *hp*EastAsia in East Asia, Oceania, and the Americas. There are additional within-regional clustering splits in *hp*Africa1 populations into western (*hsp*WAfrica) and southern (*hsp*SAfrica) subpopulations, and *hp*EastAsia into mainland East Asian (*hsp*EAsia), Oceanic (*hsp*Maori), and Native American (*hsp*Amerind) subpopulations, and a more distantly related population to all the other, hpAfrica2, only isolated in South Africans. African human populations have a low incidence of gastric cancer (upon standardized age data), which is at odds with those reported in some European and East Asian populations, despite the almost ubiquitous prevalence of *H. pylori* infection [19], a phenomenon denoted as the “African enigma” [20]. These differences were also observed between ethnicities living close by in admixed Latin America, more precisely in Colombia [21]: a low-risk coastal community of admixed African, European, and Amerindian ancestries; a high-risk Andean population mainly of Amerindian ancestry, with a minority of European ancestry. The authors verified that the severity of gastric disease correlated with the proportion of African *H. pylori* ancestry in patients with primarily Amerindian ancestry (mismatched ancestries), while patients with a large proportion of African human ancestry infected by African *H. pylori* strains (matched African ancestries) had the best prognosis. The mismatch between *Helicobacter* and *Homo* ancestries accounted for the difference in disease risk between the two communities, whereas the *H. pylori* virulence factor CagA did not. Thus, these findings were consistent with the idea that neither human nor *H. pylori* genetic variation can confer susceptibility or virulence per se, making it necessary to consider these together [21].

Despite the likely etiological importance of human–pathogen coevolution, attempts at laboratory confirmation have been rare. In the context of *H. pylori* infection, only one in vitro study has been conducted using strains of European and African ancestry to infect the Caucasian-derived AGS human cell line [22]. The authors showed that European strains promoted significantly higher host cell *IL8* expression than African strains, whereas African strains promoted host cell apoptosis. Although these findings support the influence of *H. pylori* ancestry in promoting gastric disease, they did not precisely inform about the molecular mechanisms regulating coevolution.

In this work, we propose to shed light on the coevolution theory by investigating which human genes and pathways are involved in specific interactions between host and pathogen, under dual matched and mismatched ancestry conditions. Given the theoretical expectation of stronger adaptation for the African host and pathogen organisms, we focused on comparing the dual African and European ancestries. For that, and after selecting human gastric cell lines and *H. pylori* strains of African and European ancestries, we conducted matched and mismatched in vitro coinfection assays, evaluated complete human gene expression profiles and functionally assessed the cytotoxicity, viability, apoptosis, oxidative stress and lactate production in those settings.

## 2. Materials and Methods

### 2.1. Ancestry Inference of the Gastric Cell Lines

Ancestry was inferred for 41 gastric cell lines whole exome sequenced as part of the Cancer Cell Line Encyclopedia (CCLE; [23]). The whole genome sequence information for the worldwide populations from the 1000 Genomes Project [24] was used as reference for ancestry inference. GATK HaplotypeCaller (version 3.7) was used for variant calling of these two sets and after merging common variants (amounting to 173,128 single nucleotide polymorphisms; SNPs), these were pruned for pairwise linkage disequilibrium (LD) in PLINK [25], by removing any SNP that had an r^2^ > 0.2 with another SNP, within a 50-SNPs sliding window with a step of 10 SNPs (final count of 58,537 SNPs). ADMIXTURE [26] was used to infer genetic structure of the pruned merged dataset in K = 3 ancestry components, representing the main population groups from Africa, Europe and Asia.

### 2.2. Gastric Cell Culture and Bacterial Growth Conditions

The human gastric cancer cell lines Hs746T (C0023003; AddexBio, San Diego, CA, USA), MKN74 (a kind gift from Carla Oliveira, University of Porto) and NCI-N87 (ATCC^®^ CRL-5822^TM^; ATCC, Manassas, VA, USA), referred as *Hs*EUR, *Hs*EAS and *Hs*AFR, respectively, were cultured in DMEM with Glutamax (Gibco, Dublin, Ireland) supplemented with 10% fetal bovine serum (LabClinics, Barcelona, Spain) and 100 U-100 µg/mL penicillin-streptomycin sulfate (Gibco, Dublin, Ireland), at 37 °C, under a 5% CO_2_ humidified atmosphere.

*H. pylori* strain 26695 (ATCC 700392) was obtained from ATCC while *H. pylori* strain J99 (ATCC 700824) was a kind gift from Celso Reis, University of Porto. These strains will be further referred as *Hp*EUR and *Hp*AFR, respectively. They were cultured in Trypticase^TM^ Soy Agar with 5% Sheep Blood (TSAII; Becton, Dickinson and Company, Franklin Lakes, NJ, USA) at 37 °C under microaerophilic conditions (GENbox microaer; bioMérieux S.A., Marcy-l’Étoile, France) for 48 h.

### 2.3. Infection of Gastric Cells

Gastric cancer cell lines were grown in antibiotic-free medium at approximately 80% confluence in 75 cm^2^ tissue culture flasks (VWR, Radnor, PA, USA). Medium changes were carried out every other day and immediately before infection. For infection experiments, bacteria grown for 48 h were collected in phosphate buffer saline (PBS; pH 7.4) and added to gastric cell monolayers, at a multiplicity of infection (MOI) of 100 bacteria per cell. Coinfection of the selected gastric cell lines with the different *H. pylori* strains is schematized in Supplementary Materials, Figure S1. Co-cultures were maintained for 24 h at 37 °C, under a 5% CO_2_ humidified atmosphere. Uninfected control cell cultures were processed similarly, with the addition of PBS without bacteria. Three biological replicates were performed per infection and per gastric cancer cell line.

### 2.4. Human RNA Processing and AmpliSeq Expression Profiling

mRNA was extracted from the samples with Trizol (Life Technologies, Carlsbad, CA, USA) according to the manufacturer’s protocol and quantified by spectrophotometry on an Agilent 2100 Bioanalyzer (Agilent, Santa Clara, CA, USA). Quality of samples was checked through Qubit 3.0 fluorometer and Qubit RNA HS Assay kit (ThermoFisher Scientific, Waltham, MA, USA) in the Agilent 2100 Bioanalyzer (Agilent, Santa Clara, CA, USA). Reverse transcription of RNA was done using the SuperScript^®^ VILO™ cDNA Synthesis Kit (ThermoFisher Scientific, Waltham, MA, USA). Target transcriptome sequencing was performed with the Ion AmpliSeqTM Transcriptome Human Gene Expression Kit (ThermoFisher Scientific, Waltham, MA, USA), which contains a 150 bp amplicon for each of 20,802 human genes, and quality control was checked in an Agilent 2200 TapeStation (Agilent, Santa Clara, CA, USA). The template preparation was done in an Ion Chef^TM^ System with the Ion 550™ Chip Kit, and sequencing was performed in an Ion S5TM XL System (ThermoFisher Scientific, Waltham, MA, USA). FASTQ files were generated and aligned using the Torrent Suite™ Software against the Ion AmpliSeq^TM^ Transcriptome target region (GRCh37 human reference) to infer the human expression profiles.

### 2.5. Cell Viability, Cytotoxicity and Apoptosis Assays

Gastric cancer cells were seeded in 96 well plates (BD Biosciences, San Jose, CA, USA) for two days to guarantee a density of 20,000 cells per well. The coinfection of these cells was carried out following the specifications previously described, maintaining the 24 h as period of infection. ApoTox-Glo^TM^ triplex assay (Promega, Madison, WI, USA) was then used to measure apoptosis, cell viability and cytotoxicity. An amount of 20 μL of viability/cytotoxicity reagent containing both GF-AFC and bis-AAF-R110 substrates was added to all wells, and briefly mixed. GF-AFC, glycyl-phenyl-alanyl-aminofluorocoumarin, enters in intact cells where it is cleaved by the live-cell protease activity to generate a fluorescent signal proportional to the number of living cells. Bis-AAF-R110, bis-alanylalanyl-phenylalanyl-rhodamine 110 is not cell-permeant, hence, no signal from this substrate is generated by intact, viable cells, being instead transformed by dead-cell proteases which were released from cells that have lost membrane integrity. After incubation for 1 h at 37 °C, the live- and dead-cell proteases produce AFC and R110 products, respectively, which have different excitation and emission spectra, allowing their simultaneous detection in a Synergy microplate reader (BioTek, Winooski, VT, USA): cell viability was measured by fluorescence at 400_Ex_/505_Em_, while cytotoxicity was estimated at 485_Ex_/520_Em_. For analysis of apoptosis, 100 μL of Caspase-Glo^®^ 3/7 reagent was then added to all wells and incubated for 1 h at room temperature. Addition of this reagent results in cell lysis, followed by caspase cleavage of the substrate and generation of a luminescent signal produced by luciferase that was measured in the same reader. Three biological replicates were performed per infection and per gastric cancer cell line. As positive controls of viability/cytotoxicity and apoptosis, respectively, 100 µM ionomycin (Sigma-Aldrich, St. Louis, MO, USA) which is toxic to the cells and 10 µM of staurosporine (Sigma-Aldrich) that causes apoptosis, were incubated for 6 h before addition of the assay compounds.

### 2.6. L-Lactate Concentration and Reactive Oxygen Species (ROS) Production Assays

Gastric cancer cell lines were grown in antibiotic-free medium at approximately 500,000 cells in 6 wells plates for two days. Infection was carried out for 24 h following the specification previously described. After this period, culture medium was removed, filtered by a 0.22 µm cellulose filter (Frilabo, Maia, Portugal), and stored at −80 °C after deproteinization with perchloric acid and neutralization by KOH, for further measurement of L-Lactate concentrations, using the Lactate Assay Kit (MAK064; Sigma-Aldrich, St. Louis, MO, USA). Briefly, 40 μL of the Master Mix were added to each well with 40 μL of deproteinized culture medium (either uninfected or infected) and incubated for 30 min at room temperature, protected from light. This enzymatic assay resulted in a colorimetric product that was measured in a Synergy microplate reader at 570 nm (BioTek, Winooski, VT, USA), whose intensity is proportional to the L-Lactate concentration in the sample. A calibration curve was constructed with 0, 2, 4, 6, 8 and 10 nmol/well of the standard solution. Concentration values were normalized for the amount of cells per sample. Three biological replicates were used per infection and per gastric cancer cell line.

New culture medium was added to the adherent cells (post-infection) which were used to estimate mitochondrial superoxide (the most abundant ROS) production, through MitoSOX^TM^ Red (Invitrogen, Carlsbad, CA, USA). MitoSOX™ Red reagent is a fluorogenic dye specifically targeted to mitochondria in live cells, and its oxidation by superoxide produces red fluorescence. Briefly, 2.5 µM MitoSOX were added to each well, and incubated for 30 min at 37 °C, protected from light. Culture medium was then removed, and adherent cells were treated with 0.25% trypsin-EDTA (Invitrogen 25,200, Carlsbad, CA, USA) and collected separately into a microcentrifuge tube. Cells were then washed once with PBS and centrifuged at 300× *g*. MitoSOX fluorescence was measured by flow cytometry at 488 nm (excitation) and 575 nm (emission) using the BD Accuri C6 Plus (BD Biosciences, San Jose, CA, USA). Results were analysed by FlowJo v10.0.7 (Tree Star, Inc., Ashland, OR, USA) using the mean fluorescence intensity values for the FL2 channel. Three biological replicates were used per infection and per gastric cancer cell line. Cell controls without MitoSOX probe and with MitoSOX with 50 µM antimycin (Sigma-Aldrich, St. Louis, Missouri, USA; that causes oxidative stress) treatment were used as negative and positive controls, respectively.

### 2.7. Algorithms and Statistics Applied to Gene Expression and Functional Data

All the statistical analysis and graphical representations presented in this work were performed in R version 3.6 [27]. Quality control checks of expression profiles of triplicates were investigated through Principal Component Analysis (PCA) and the rooted neighbor-joining tree, based on a matrix of Euclidean genetic distance, was obtained from the pruned whole exome variability from the 41 gastric cancer cell lines.

Differentially expressed genes between paired tests were identified by DESeq2 package [28], which applies a negative binomial distribution test (adjusted *p*-value threshold below 0.05 was considered). Clustering and heatmap representations of these significantly expressed genes, between uninfected and infected tests, were obtained using heatmap.3 package.

Pre-ranked pairwise gene-set enrichment analyses (GSEA) were conducted in GSEA-InContext [29] for the GO-biological process ontology. Results were ordered by the normalized enrichment score (NES) and the false discovery rate (FDR). Specific significantly enriched pathways were further explored based on information contained in the publicly available database of Ingenuity (https://targetexplorer.ingenuity.com/index.htm; Qiagen, Hilden, Germany). The list of intervening genes and chemical reactions was collected and used in drawing schematic representations containing information of significant fold-changes in expression levels when comparing the infected versus non-infected experimental settings.

In order to get a focused insight on the innate immune response to the infection, the InnateDB [30] was used, amounting to 951 protein-coding genes having a role in the innate immune response.

Welch’s t-tests were applied in R to compare the mean values of cell viability, cytotoxicity, apoptosis, L-Lactate concentration and ROS production, and values below 0.05 were considered significant. The Welch’s t-test adjusts the number of degrees of freedom when the variances are unequal.

## 3. Results

### 3.1. Ancestry of the Gastric Cell Lines and H. pylori strains

**Figure 1:**
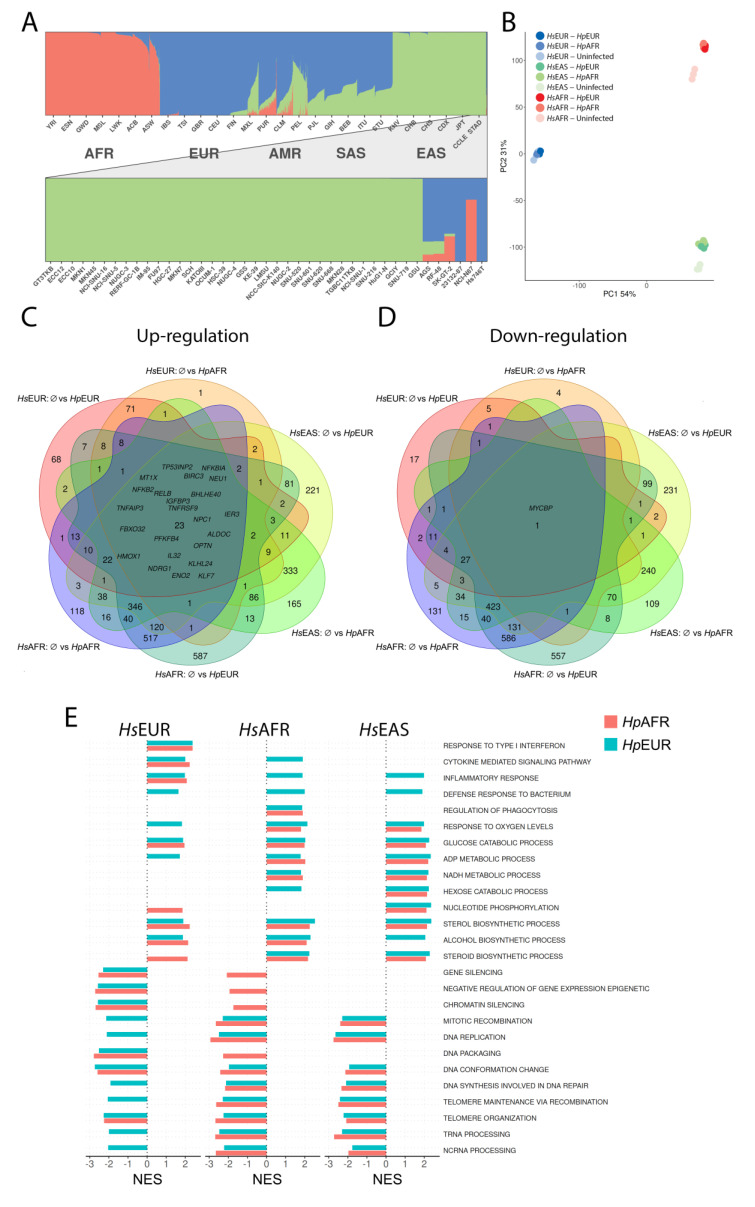
Ancestry profiles of human gastric cancer cell lines and genome-wide expression profiles. (**A**)—Population structure inferred by ADMIXTURE analysis of 1000 Genomes populations and CCLE panel, in which each individual is represented by a vertical (100%) stacked column of genetic components proportions shown in color for K = 3 (AFR—African ancestry in red, EUR—European in blue and EAS—Asian in green; AMR and SAS are, respectively, American and South Asian populations). Lower plot is a zoom of the CCLE panel. (**B**)—PCA plot (PC1 explaining 54% of variation vs. PC2 explaining 31% of variation) of the human transcriptomic profile of the three gastric cell lines (triplicates for each condition; blue colors for the European; green for the Asian and pink for the African) without and after infection with *Hp*AFR and *Hp*EUR *H. pylori* strains. (**C**)—Venn diagrams for up-regulated and (**D**)—down-regulated genes in all experimental *H. pylori* infected settings compared with the uninfected status (indicated by Ø). (**E**)—Top significantly enriched pathways in pairwise comparisons between infected (positive side) and uninfected (negative side) conditions in the three cell lines. The different color represents *H. pylori* strains (pink for African and blue for European ancestries). The scale reports the normalized enrichment score (NES) values.

Ancestry information was not available for most of the stomach cancer cell lines at the time we began this work. We selected the 41 cancer cell lines exome sequenced as part of the Cancer Cell Line Encyclopedia (CCLE, [23]) and merged these samples with the populations from the 1000 genomes project [24]. Admixture analysis (Figure 1A, Supplementary Table S1) revealed that the great majority of the cell lines (35 out of 41) derive from an East Asian (EAS) background. Only two cell lines (Hs746T and 23132-87) were isolated from individuals of European ancestry. One cell line harbored 75% African and 25% European backgrounds (NCI-N87), probably derived from an admixed African-American individual (according to the mean 27% and 22% European input in the African-American cohorts of northern and southern USA, respectively [31]). The remaining three cell lines had varying degrees of admixture between the three ancestries. Based on these results, we selected the only option for *Hs*AFR (NCI-N87), and between the two available options for the *Hs*EUR, we selected the one with fewer mutations (Hs746T with 608 mutations according to CCLE website, instead of 23132-87 with 3150 mutations; for comparison, NCI-N87 has 477, and MKN74 has 738 mutations). Additionally, we selected the widely used *Hs*EAS (MKN74) for infection assays with European (close mismatch) and African (distant mismatch) *H. pylori*.

Meanwhile, Dutil et al. [32] published their ancestry inference in the entire CCLE panel, based on another technology, the Affymetrix SNP6.0 chip. Their results, also using ADMIXTURE but ascertaining information for seven ancestry components (African, Native American, North and Southeast Asian, South Asian, and North and South European) inferred the following proportions of the cell lines used here: *Hs*EUR (Hs746T)—96.36% European, 3.48% Asian (East and South), and 0.16% Native American; *Hs*AFR (NCI-N87)—61.23% African; 36.60% European and 2.17% Asian; and *Hs*EAS (MKN74)—98.71% Asian and 1.29 European. These values are largely concordant with our inference for *Hs*EUR and *Hs*EAS. However, the differences are higher (by 14%) for the proportion of African ancestry inferred for the *Hs*AFR. We downloaded their data and confirmed their proportions when running an ADMIXTURE for K = 3, which leads us to suggest the high European-prone ascertainment biases introduced by the chip used in Dutil et al. [32] as the best cause for this discrepancy when compared to the non-ancestry biased whole exome sequencing. Despite these incongruities, the *Hs*AFR cell line has a dominant African ancestry.

Regarding the *H. pylori* strain selection, we followed published phylogeographic data [33] and selected *H. pylori* J99 of West African ancestry and *H. pylori* 26695 of European ancestry, henceforth abbreviated as *Hp*AFR and *Hp*EUR, respectively. These strains are both CagA-positive and have the toxic form VacA.

### 3.2. Global Expression Profile Alterations

The PCA for the overall expression profile (Figure 1B) shows, as expected, that the cell line background is the main clustering factor. The three cell lines are almost equidistant in terms of their expression profiles, despite the closeness between *Hs*EUR and *Hs*EAS in terms of exome diversity (Supplementary Figure S2). Another important information from the PCA is the clear distinction between the expression profiles of uninfected versus infected (either matched or mismatched) status for *Hs*EAS and *Hs*AFR, and less so for *Hs*EUR.

Differentially expressed genes between uninfected and infected conditions showed statistically significant (adjusted *p* < 0.05) alterations in gene expression (Supplementary Tables S2–S7): in *Hs*EUR, 515 and 209 were up-regulated, and 364 and 31 were down-regulated when infected by *Hp*EUR and *Hp*AFR, respectively; in *Hs*AFR, those numbers were 2335 and 1554, and 2279 and 1745; and in *Hs*EAS, those numbers were 2554 and 2214, and 2464 and 2159.

Twenty-three genes were up-regulated (log2-fold change > 1) in common to all infection settings, mainly related to I-kappaB kinase/NF-kappaB signaling (adjusted *p* = 0.0015; Figure 1C). The *MYCBP* gene, which controls the transcriptional activity of the proto-oncogene *MYC* and that plays a role in cell cycle progression, apoptosis and cellular transformation, was the only gene down-regulated in common to all infection settings (Figure 1D).

We also checked for cell line specific common alterations to infection by each strain, which could indicate specific human ancestry response to infection. Upon infection, we observed 71, 517 and 333 up-regulated genes in *Hs*EUR, *Hs*AFR and *Hs*EAS, respectively (Figure 1C). The enrichment analysis by g:Profile of these genes revealed their involvement in different pathways: type I interferon signaling pathway (*p* = 4.1 × 10^−18^) in *Hs*EUR; pathways related to cellular metabolic processes, such as cellular response to endogenous stimulus (*p* = 6.6 × 10^−4^), intracellular signal transduction (*p* = 1.7 × 10^-3^) and protein metabolic processes (*p* = 1.9 × 10^-3^) in *Hs*AFR; and, *MAPK* (*p* = 7.0 × 10^−5^), *RAS* (*p* = 1.0 × 10^−3^) and *RAP1* signaling pathways (*p* = 2.6 × 10^−2^) in *Hs*EAS. Regarding common down-regulated genes, they were involved in: no signal in *Hs*EUR (only 5 genes); metabolic pathways such as ribosome biogenesis (*p* = 1.5 × 10^−24^) and cellular metabolic process (*p* = 7.5 × 10^−12^) in *Hs*AFR (586 genes); and positive regulation of apoptotic process (*p* = 2.6 × 10^−3^) in *Hs*EAS (240 genes).

In summary, this global expression pattern shows that *H. pylori* infection, irrespectively of matched or mismatched ancestries, activates immune-related I-kappaB kinase/NF-kappaB signaling, and down-regulates the cell cycle related pathways of the human gastric cells. Additional cell line specific pathways are activated upon infection, and their involvement in a differential human ancestry cellular response to infection was further explored.

### 3.3. Detailed Analysis of Altered Molecular Pathways

Molecular pathways are highly complex, with multiple redundant control systems. Therefore, the additive effect of small changes in expression of several genes can better describe the complex biological systems (and have higher statistical power) than an extreme fold change in expression of one or two genes. This is the rationale behind gene set enrichment analysis (GSEA), which we applied to the pairwise comparisons for each experimental setting. Figure 1E summarizes the top pathways (Supplementary Tables S8–S16 report all results). For the *Hs*EUR, the comparison between uninfected vs. infected (both *H. pylori* strain ancestries) revealed top enrichment of immune-related pathways (and at the first position, the response to type I interferon) and top down-regulation of cell cycle and DNA repair pathways. These enrichment hits remained the main significant result when comparing matched (*Hs*EUR × *Hp*EUR) versus mismatched (*Hs*EUR × *Hp*AFR) ancestry sets. In *Hs*AFR, infection in general increased especially lipid metabolism, and down-regulated cell cycle, while the matched-mismatched conditions led to top enrichment of response to oxidative stress in the mismatched (*Hs*AFR × *Hp*EUR) and cilium morphology and protein transport in the matched setting (*Hs*AFR × *Hp*AFR), respectively. Concerning the *Hs*EAS, the pairwise comparison between uninfected and infected status revealed a major enrichment of metabolic pathways related to the production of energy, and down-regulation of cell cycle pathways. Infection with *Hp*EUR in *Hs*EAS (close-mismatch) led to enrichment of immune-related pathways whereas infection with *Hp*AFR (distant-mismatch) led to enrichment of pathways related to blood vessels and wound healing.

The GSEA confirmed the up-regulation of NF-kappaB related pathways in all infection sets, especially significant in *Hs*EUR (FDR = 0.0027 for *Hp*EUR; 0.0015 for *Hp*AFR) than in *Hs*EAS (0.071 for *Hp*EUR; 0.65 for *Hp*AFR) and *Hs*AFR (0.053 for *Hp*EUR; and not statistically significant, 0.16, for *Hp*AFR). Statistically significant up-regulation of response to type I interferon was observed in *Hs*EUR (0 for infections with *Hp*EUR and *Hp*AFR).

### 3.4. Exploring the Enriched Pathways—Innate Immune System

**Figure 2:**
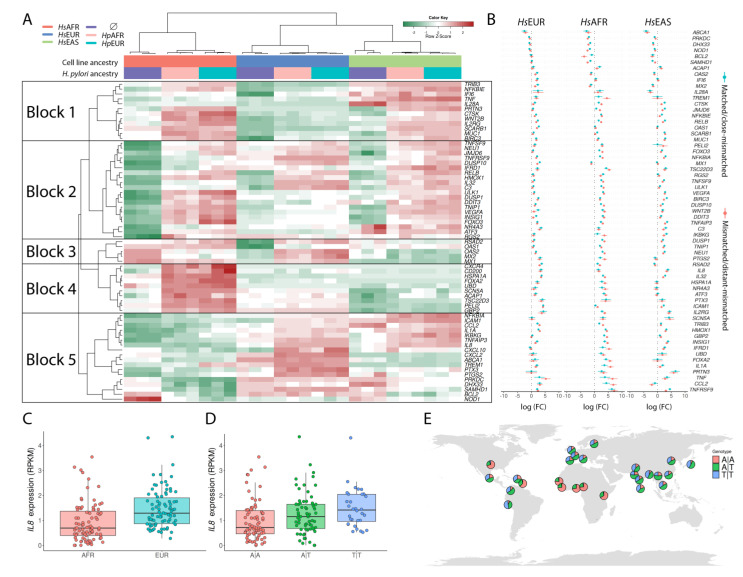
Expression profiles for innate immune system genes based on InnateDB database and *IL8* variability. (**A**)—Clustering and heat map of the most significant differences in expression (in any pairwise comparison of infection versus uninfected) in at least one of the three cell lines. (**B**)—Log2 changes in expression of gene represented in A when infections were conducted with matched (for *Hs*EUR and *Hs*AFR) or close-mismatch (for *Hs*EAS) bacteria in blue, and between mismatched (for *Hs*EUR and *Hs*AFR) or distant-mismatch (for *Hs*EAS) bacteria in red. (**C**)—*IL8* gene expression profiles in African (Yoruban population; in pink) and European (Great Britain population; in blue) in the RNAseq data available in the 1000 Genomes consortium website. (**D**)—*IL8* gene expression profiles depending on the genotypes at *rs4073* SNP (AA in pink; AT in green; TT in blue). (**E**)—Genotype frequency distributions (same colors as in **B**) across the globe as inferred from 1000 Genomes populations.

Considering that our experimental infection model only includes gastric epithelial cells we focused on innate immune response genes, by referring to the InnateDB database [30], which includes 951 curated protein-coding genes involved in this response. Figure 2A represents the most significant differences in expression (in any pairwise comparison) in at least one cell line. The first clustering of genes (block 1) is, in general, overexpressed with infection in *Hs*EAS and *Hs*AFR, but not in *Hs*EUR. These genes are involved in the regulation of phagocytosis (*p* = 4.0 × 10^−4^) and cytokine-mediated signaling (*p* = 4.0 × 10^−3^). Genes in block 2 are, in general, overexpressed in cell lines of all ancestries upon infection, and are related to negative regulation of the *MAPK* cascade (*p* = 1.0 × 10^−3^). Genes in block 3 are up-regulated upon infection in particular in *Hs*EUR, and are related to type-1 interferon signaling pathway (*p* = 4.7 × 10^−7^). Further evaluation of the genes involved in this pathway showed that *IFNAR1*, *JAK1*, *TYK2*, *STAT1*, *STAT2* and *IRF9* are up-regulated in *Hs*EUR upon infection, while *STAT1*, *STAT2* and *IRF9*, members of the interferon-stimulated gene factor 3 (*ISGF3*), were down-regulated during infection in both *Hs*EAS and *Hs*AFR experimental settings (Supplementary Figure S3). Genes in block 4 are mainly up-regulated by the infection in *Hs*AFR, and are associated with endocytosis (*p* = 2.5 × 10^−2^) and response to cytokine (*p* = 2.1 × 10^−2^). Genes in block 5 are mainly unaffected by the infection in *Hs*AFR, and these genes are related to IL-17 signaling pathway (*p* = 2.0 × 10^−10^), NF-kappa B signaling pathway (*p* = 4.4 × 10^−10^), NOD-like receptor signaling pathway (*p* = 7.2 × 10^−10^) and TNF signaling pathway (*p* = 1.1 × 10^−9^). No major differences in expression levels of these innate immune genes were observed in the host cell lines of different ancestries upon infection with bacteria of matched and mismatched ancestries (Figure 2B).

The chemokine *IL8*/*CXCL8* (in block 5) deserves a more careful inspection, as some of its SNPs have been associated, although disputably, to risk of gastric cancer [34]. This gene is up-regulated upon infection in the European and East Asian cell lines, but it is consistently expressed at low levels in both the uninfected and infected settings of the African cell line. We compared the expression of this gene between European (Great Britain) and African (Yoruba) in the RNASeq data available in the 1000 Genomes consortium website and confirmed that the gene is significantly (*p* = 2.0 × 10^−7^) less expressed in the latter than in the former population (Figure 2C). When attending to the genomes of the associated SNP, *rs4073* (formally designated as −251 T/A), the expression of the gene (Figure 2D) decreases with the dose of allele A (adjusted *p*-values: AA vs. AT—0.0335; AA vs. TT—0.0023; AT vs. TT—0.1541). This SNP presents (Figure 2E) a high heterogeneity between population groups. The low-expressing *IL8* AA genotype is predominant in African populations (~70% frequency) and has a low frequency in European and East Asian populations (<20%). The genotypes for *rs4073* of the cell lines used in this study are AA for both *Hs*AFR and *Hs*EAS, and TT for *Hs*EUR.

### 3.5. Exploring the Enriched Pathways—Lactate Metabolism

**Figure 3:**
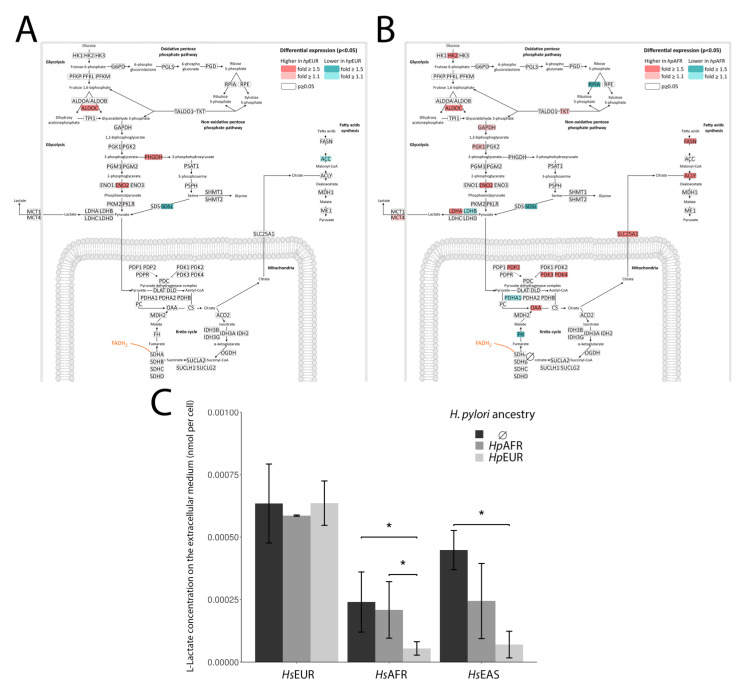
Metabolism of L-Lactate. (**A**)—Statistically significant fold changes (up-regulation in pink and down-regulation in blue) in gene expression for the *Hs*EUR × *Hp*EUR setting versus uninfected in the glycolysis, Krebs cycle and fatty acids synthesis. (**B**)—Statistically significant fold changes (up-regulation in pink and down-regulation in blue) in gene expression for the *Hs*AFR × *Hp*AFR setting versus uninfected in the glycolysis, Krebs cycle and fatty acids synthesis. (**C**)—Extracellular lactate concentration in uninfected and infected settings in the three cell lines (mean and standard deviations of triplicates). Statistically significant comparisons are indicated by *.

Few studies addressed the metabolic effects of *H. pylori* infection on gastric epithelial cells [35,36,37]. *H. pylori* use L-lactate released by gastric epithelial cells to obtain growth benefits [37]. The bacterial genes encoding the L-lactate dehydrogenase were recently identified [35]. Our data showed that infection is associated with enrichment in gene expression of *LDHA* (production of L-lactate in the host cell) and *SLC16A4*/*MCT4* (export of L-lactate from the host cell) in *Hs*EAS and *Hs*AFR (Figure 3A,B and Supplementary Figures S4–S6), and with a significant decrease of L-lactate concentration in the extracellular culture medium of the infected settings (Figure 3C). These results endorse the idea that *H. pylori* stimulate the secretion of L-lactate by gastric cells for its benefit. Regarding the *Hs*EUR cell line, no significant differences were observed for the L-lactate concentrations between the uninfected and the infected settings. A possible explanation for this observation is the a priori high concentration of L-lactate in the *Hs*EUR cell line compared with the other two (Supplementary Figure S7, based on metabolome data from [38]), allowing a higher amount of L-lactate released to the extracellular medium readily available to *H. pylori*. In terms of lactate metabolism stimulation by the bacteria, there were no major differences between matched and mismatched infections, except in *Hs*AFR infected by *Hp*EUR.

Further evaluation with available data on human and bacteria gene expression profiles (PRJNA378649; no associated publication yet) from co-infection experiments with the same cell line as our *Hs*AFR and *Hp*EUR bacterial strain allowed us to ascertain the significant increase in expression of two out of the three lactate-related bacterial genes (HP0137—1.6 log2 fold change; and HP0138—0.2 log2 fold change).

### 3.6. Exploring the Enriched Pathways—Mitochondrial Oxidative Stress

**Figure 4:**
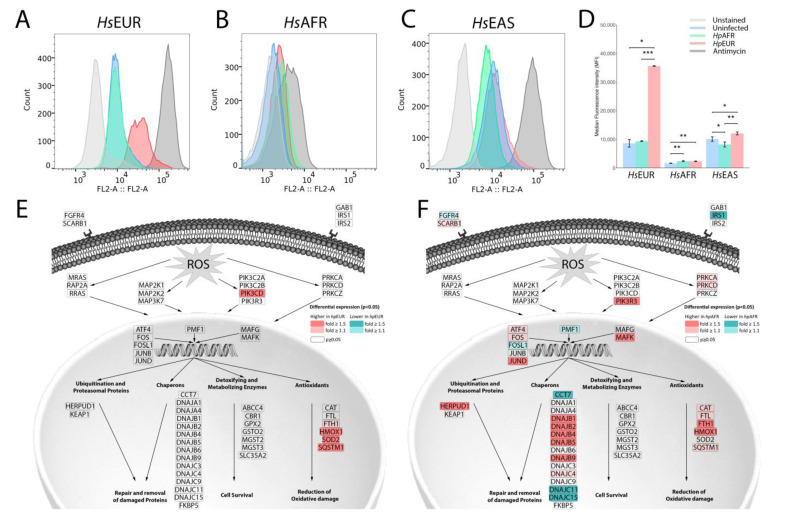
Reactive oxygen species (ROS). (**A**–**C**)—MitoSOX fluorescence values for the various settings in each cell line. (**D**)—Mean values and standard deviations of fluorescence values for the various settings in each cell line. (**E**)—Statistically significant fold changes (up-regulation in pink and down-regulation in blue) in gene expression for the *Hs*EUR × *Hp*EUR setting versus uninfected in the response to ROS pathway. (**F**)—Statistically significant fold changes (up-regulation in pink and down-regulation in blue) in gene expression for the *Hs*AFR × *Hp*AFR setting versus uninfected in the response to ROS pathway. Statistically significant comparisons are indicated by *. * means: *p* < 0.05, ** means: *p* < 0.01, *** means: *p* < 0.001.

*H. pylori* infection in humans is associated with enhanced levels of reactive oxygen species (ROS), increased oxidative DNA damage, and diminished glutathione in the mucosa [39]. Based on this, we examined whether *Hp*AFR and *Hp*EUR elicited different ROS production in the different host cells and whether these results were linked to specific cellular responses associated with oxidative stress. ROS levels (Figure 4A–D) were significantly increased by infection with *Hp*EUR in all cell lines compared to the uninfected controls, with the *Hs*EUR cell line being the most affected (250% increase). In contrast, infection with *Hp*AFR had irregular effects in the different cell lines (no differences in *Hs*EUR, increase in *Hs*AFR and reduction in *Hs*EAS).

Analysis of the differentially expressed genes associated with oxidative stress revealed that infection in all cell lines significantly activated genes related to antioxidants, most probably as a means to reduce the oxidative damage (Figure 4E,F and Supplementary Figures S8–S10). Interestingly, while in *Hs*EUR, only that part of the pathway was showing the expression changes, in both *Hs*AFR and *Hs*EAS there were also alterations in chaperon and ubiquitination proteins related with repair and removal of damaged proteins (mostly up-regulation, in particular in *Hs*AFR), and in the detoxifying and metabolizing enzymes associated with cell survival (specially down-regulation when *Hs*AFR was infected by *Hp*EUR). These results might indicate that *Hs*AFR and *Hs*EAS, have a more efficient response to ROS than *Hs*EUR, which might explain the higher concentration of ROS displayed by this cell line upon infection with *Hp*EUR.

**Figure 5:**
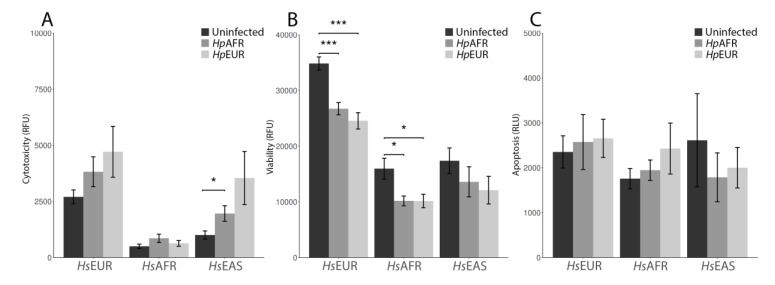
Mean and standard deviation values for cellular cytotoxicity (**A**), viability (**B**) and apoptosis (**C**) in each cell line infected for 24 h. Statistically significant comparisons are indicated by *. * means: *p* < 0.05, *** means: *p* < 0.001.

Concerning the well-established relationship between oxidative stress and cellular damage, we investigated the impact of the different ancestries in cellular viability, cytotoxicity, and apoptosis in each cell line infected for 24 h (Figure 5; *t*-test results are listed in Supplementary Table S17). The infection decreased cell viability of the *Hs*EUR, *Hs*AFR and *Hs*EAS cell lines, although in the latter the decrease was not statistically significant. Cytotoxicity was negatively associated with viability. Quantification of caspase 3/7 luminesce for the presence of apoptosis revealed that *H. pylori* did not induce any significant differences in the level of apoptosis.

## 4. Discussion

By applying a genome-wide approach to the host transcriptome, supported by some functional confirmations, in controlled double-ancestry host–pathogen in vitro settings, we were able to provide experimental evidence to address three main questions: (1) do the host responses to *H. pylori* infection, in terms of whole-genome expression profile, differ between human ancestries; (2) are coevolution model expectations fulfilled, with higher adaptation of the African ancestry to *H. pylori* infection, and maladaptation of European and Asian populations groups; and (3) do infections caused by a mismatched *H. pylori* ancestry differ from a matched setting?

Our results clearly show that a higher number of genes were significantly changed upon infection (up- and down-regulated) in the African and Asian cell lines than in the European (≈5 to 10 times), indicating a more complex response (possibly meaning better adaptation) to the infection stimulus. Within each cell line, numbers were of the same order of magnitude for both *Hp*EUR and *Hp*AFR infections, although slightly lower for the latter. More interesting, the top activated pathways differed between human ancestries: African up-regulated lipid metabolism, while Asian activated energy production, contrary to European which relied on immune-related pathways as the most up-regulated. All ancestries displayed down-regulation of pathways related to the cell cycle. These results seem to indicate that African and Asian ancestries had a broader, and probably better, adapted molecular response to infection by *H. pylori*, while European ancestry relied substantially on the immune system (and the signal is stronger when this cell line is infected by *Hp*EUR than *Hp*AFR). In agreement with the lipid metabolism activation in the African ancestry upon infection with *H. pylori*, we recently showed that lipid metabolism plays a major role in naturally protecting the African human ancestry from the worse phenotypes of dengue virus disease [40]. Differences in the cholesterol efflux regulatory protein (encoded by *ABCA1* gene) were already observed in *H. pylori* infection, which depletes cholesterol in gastric glands to prevent interferon-gamma signaling and to escape the inflammatory response [41].

Mucosal epithelial cells are a defense barrier against invading pathogens such as bacteria. They express pattern recognition receptors that play important roles in the initiation, maintenance, and regulation of both innate and adaptive immune responses [42], although in the context of *H. pylori* infection the elicited immune response does not lead to bacterial clearance [43]. *H. pylori* infection of the gastric mucosa induces T helper type 1 (Th1) and Th17 adaptive immune responses, as well as innate immune responses that have been less studied. The main rule of thumb was human ancestries being associated with very diverse innate immune responses. In a host with European ancestry, *H. pylori* infection distinctly activated type I IFNs. This type of response was traditionally associated with a defense against viruses but has recently been shown to be involved in bacterial infections, having protective or detrimental roles depending on the bacterium [44]. In a mouse model with impairment of *ISGF3* signaling, *H. pylori* led to decreased *CXCL10* responses and increased susceptibility to infection [45]. This type I IFN up-regulation was also consistent with the up-regulation of several interferon-stimulated genes, in the *Hs*EUR cell line, such as *MX1*, *MX2*, *IFIT1*, *IFI6*, and *OAS3*. In contrast, some genes important in IL-17, NF-kappa B, NOD-like receptor and TNF signaling pathways were not up-regulated in the host cell with African ancestry upon infection, which preferentially stimulated genes involved in phagocytosis and endocytosis. *IL17* is an essential cytokine for host defense against bacteria, promoting pro-inflammatory cascades [46]. Cells triggered by microbes secrete *IL17A*, which is recognized by an *IL17* receptor [47] activating NF-kB and MAPK/AP-1 inflammatory pathways. Activation of these pathways leads to the production of pro-inflammatory cytokines, chemokines and antimicrobial peptides, which induce inflammation required for host defense [48,49]. This evidence seems to point to a decreased pro-inflammatory response in the African human epithelial cells. We also confirmed that Africans had a baseline lower IL-8 expression than Europeans and that IL-8 did not play a role in the immune response to *H. pylori* in the human African ancestry, suggesting a lower bacterial-induced inflammation in this ancestry background. Our results are instrumental in contributing information that will help to clarify contradictory published evidence.

The response to oxidative stress induced by ROS seemed to be more effective in *Hs*AFR (and in *Hs*EAS) than in *Hs*EUR, as several genes with parallel actions to antioxidants/reduction of oxidative damage were also activated in the former and not in the latter. The *Hp*EUR strain appeared to be especially inductive of ROS accumulation, reaching a massive value when infecting with the matched ancestry *Hs*EUR cell line. In the *Hs*AFR and *Hs*EAS cell lines, both strains were able to induce the production and export of lactate by the host, allowing an increased input of lactate into the bacteria. In contrast, the concentration of lactate was already high in the uninfected *Hs*EUR, which might explain the lack of changes in this metabolism upon infection.

We can thus conclude, in response to the raised questions: (1) yes, the host response to *H. pylori* was greatly molded by human ancestry; (2) the African human ancestry showed clear signs of coevolution with *H. pylori* while the European human ancestry was maladapted, with the Asian ancestry in between (but closer to the coevolved African); and (3) the mismatched host-bacterium ancestry did not appear to be an important differentiator of gene expression, at least at the initial stages of infection, as we analyzed here. *Hp*EUR × *Hs*EUR induced similar gene expression profiles as *Hp*AFR × *Hs*EUR, and the same within the African cell line. This observation does not exclude the possibility of a worse phenotype being conferred by a mismatched rather than matched in vivo infection, in more advanced stages of the process. This possibility would render our results compatible with the observations collected in Colombian admixed cohorts [21]. Future validation of these results, when other gastric cancer cell lines of Africa and European ancestries become available, is recommended. In fact, this work exemplifies the current limitations in the basic tools available to conduct oncobiology research in the African ancestry, due to the scarcity of appropriate cancer cell lines, mostly of which are derived from admixed African-Americans (mean of 25% of European ancestry). Our results preliminarily point towards the following predictions in terms of disease risk: mismatched *Hp*AFR × *Hs*EUR will present a higher risk when compared with *Hp*EUR × *Hs*EUR, while both *Hp*EUR × *Hs*AFR and *Hp*AFR × *Hs*AFR situations will present a low risk because the African cell line seems to be better adapted to *H. pylori* in general.

## Data Availability

Raw data are publicly available at the European Nucleotide Archive (ENA) with Accession Number PRJEB42638.

## Supplementary Materials

The following are available online at https://www.mdpi.com/2076-2607/9/2/240/s1, Table S1: Admixture proportions (%; K ancestral groups = 3) for each gastric cell line from the CCLE in relation with the 1000 Genomes reference superpopulations. Table S2: Differentially expressed genes (adjusted *p* < 0.05) in the *Hs*EUR cell line infected by the *Hp*AFR strain compared to the uninfected set. The table displays fold changes and adjusted *p*-values of the differentially expressed genes. Genes with a fold change values >0 are upregulated in the infected setting. Table S3. Differentially expressed genes (adjusted *p* < 0.05) in the *Hs*EUR cell line infected by the *Hp*EUR strain compared to the uninfected set. The table displays fold changes and adjusted *p*-values of the differentially expressed genes. Genes with a fold change values >0 are upregulated in the infected setting. Table S4. Differentially expressed genes (adjusted *p* < 0.05) in the *Hs*EAS cell line infected by the *Hp*AFR strain compared to the uninfected set. The table displays fold changes and adjusted *p*-values of the differentially expressed genes. Genes with a fold change values >0 are upregulated in the infected setting. Table S5. Differentially expressed genes (adjusted *p* < 0.05) in the *Hs*EAS cell line infected by the *Hp*EUR strain compared to the uninfected set. The table displays fold changes and adjusted *p*-values of the differentially expressed genes. Genes with a fold change values >0 are upregulated in the infected setting. Table S6. Differentially expressed genes (adjusted *p* < 0.05) in the *Hs*AFR cell line infected by the *Hp*AFR strain compared to the uninfected set. The table displays fold changes and adjusted *p*-values of the differentially expressed genes. Genes with a fold change values >0 are upregulated in the infected setting. Table S7. Differentially expressed genes (adjusted *p* < 0.05) in the *Hs*AFR cell line infected by the *Hp*EUR strain compared to the uninfected set. The table displays fold changes and adjusted *p*-values of the differentially expressed genes. Genes with a fold change values >0 are upregulated in the infected setting. Table S8—GSEA GO-BP results for the *Hs*EUR × *Hp*EUR versus uninfected settings. Table S9—GSEA GO-BP results for the *Hs*EUR × *Hp*AFR versus uninfected settings. Table S10—GSEA GO-BP results for the *Hs*EAS × *Hp*EUR versus uninfected settings. Table S11—GSEA GO-BP results for the *Hs*EAS × *Hp*AFR versus uninfected settings. Table S12—GSEA GO-BP results for the *Hs*AFR × *Hp*EUR versus uninfected settings. Table S13—GSEA GO-BP results for the *Hs*AFR × *Hp*AFR versus uninfected settings. Table S14—GSEA GO-BP results for the *Hs*EUR cell line comparing *Hp*AFR versus *Hp*EUR infected settings. Table S15—GSEA GO-BP results for the *Hs*EAS cell line comparing *Hp*AFR versus *Hp*EUR infected settings. Table S16—GSEA GO-BP results for the *Hs*AFR cell line comparing *Hp*AFR versus *Hp*EUR infected settings. Table S17—T-test results for the analysis of apoptosis, cellular viability and cytotoxicity. Figure S1: “Schematic representation of the co-infection experimental design. Infection was performed in three cancer cell lines, each representative of the three main human population groups (AFR—African, EUR—European and EAS—East Asian), by two *H. pylori* strains (European and African origin). Admixture percentages for each human ancestry per cell line are specified in the left-hand side. Each infection was done in triplicate. Figure S2: SNP based phylogenetic tree. Phylogenetic tree depicting the SNP distance between the 41 CCLE gastric cancer cell lines. Figure S3: Log2 changes in gene expression between *Hp*AFR and *Hp*EUR infection sets for the Type I interferon genes and Interferon stimulated genes. Figure S4: Metabolism of lactate in the *Hs*EUR cell line. Statistically significant fold changes (up-regulation in pink and down-regulation in blue) in gene expression upon infection with A-*Hp*EUR and B-*Hp*AFR strains against the uninfected set in glycolysis, Krebs cycle, and fatty acids synthesis. Figure S5: Metabolism of lactate in the *Hs*EAS cell line. Statistically significant fold changes (up-regulation in pink and down-regulation in blue) in gene expression upon infection with A-*Hp*EUR and B-*Hp*AFR strains against the uninfected set in glycolysis, Krebs cycle, and fatty acids synthesis. Figure S6: Metabolism of lactate in the *Hs*AFR cell line. Statistically significant fold changes (up-regulation in pink and down-regulation in blue) in gene expression upon infection with A-*Hp*EUR and B-*Hp*AFR strains against the uninfected set in glycolysis, Krebs cycle, and fatty acids synthesis. Figure S7: Lactate cellular concentration (normalized mass-spectrometry values; see original paper for description) amongst 37 CCLE stomach cell lines, based on metabolomic data from [38]. *Hs*EUR in blue, *Hs*EAS in green and *Hs*AFR in red. Figure S8: Illustration of the oxidative stress response in the *Hs*EUR cell line. Statistically significant fold changes (up-regulation in pink and down-regulation in blue) in gene expression upon infection with A-*Hp*EUR and B-*Hp*AFR strains against the uninfected set. Figure S9: Illustration of the oxidative stress response in the *Hs*EAS cell line. Statistically significant fold changes (up-regulation in pink and down-regulation in blue) in gene expression upon infection with A-*Hp*EUR and B-*Hp*AFR strains against the uninfected set. Figure S10: Illustration of the oxidative stress response in the *Hs*AFR cell line. Statistically significant fold changes (up-regulation in pink and down-regulation in blue) in gene expression upon infection with A-*Hp*EUR and B-*Hp*AFR strains against the uninfected set.
