# Supplementary material for: Shedding Light on the African Enigma: In Vitro Testing of Homo sapiens-Helicobacter pylori Coevolution
Source: Microorganisms. 2021 Jan 25;9(2):240. doi: 10.3390/microorganisms9020240 (PMC7912213; doi:10.3390/microorganisms9020240)
Supplement: Supplementary file 1 [file microorganisms-09-00240-s001.zip › microorganisms-1056872-sl-prood done/microorganisms-1056872.SupplementaryFigures.docx]

**Supplementary figures**


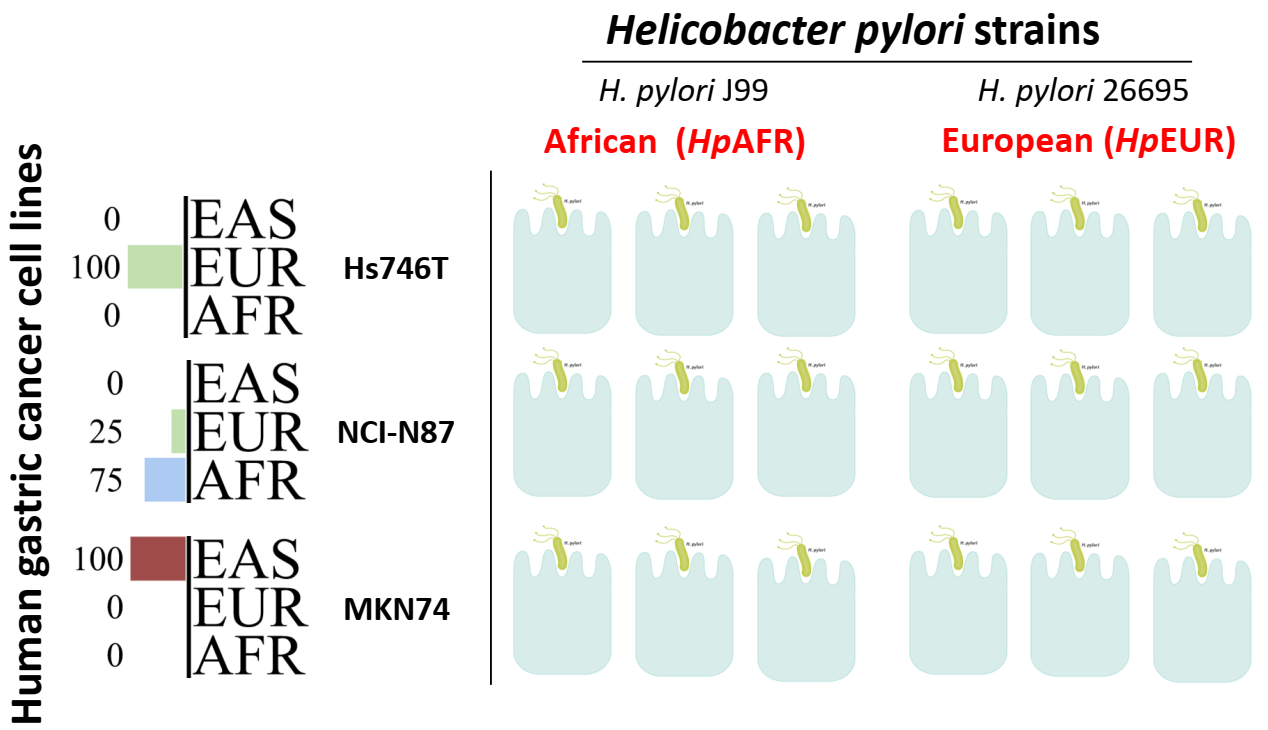


Supplementary Figure S1- “Schematic representation of the co-infection experimental design. Infection was performed in three cancer cell lines, each representative of the three main human population groups (AFR-African, EUR-European and EAS-East Asian), by two *H. pylori* strains (European and African origin). Admixture percentages for each human ancestry per cell line are specified in the left-hand side. Each infection was done in triplicate.


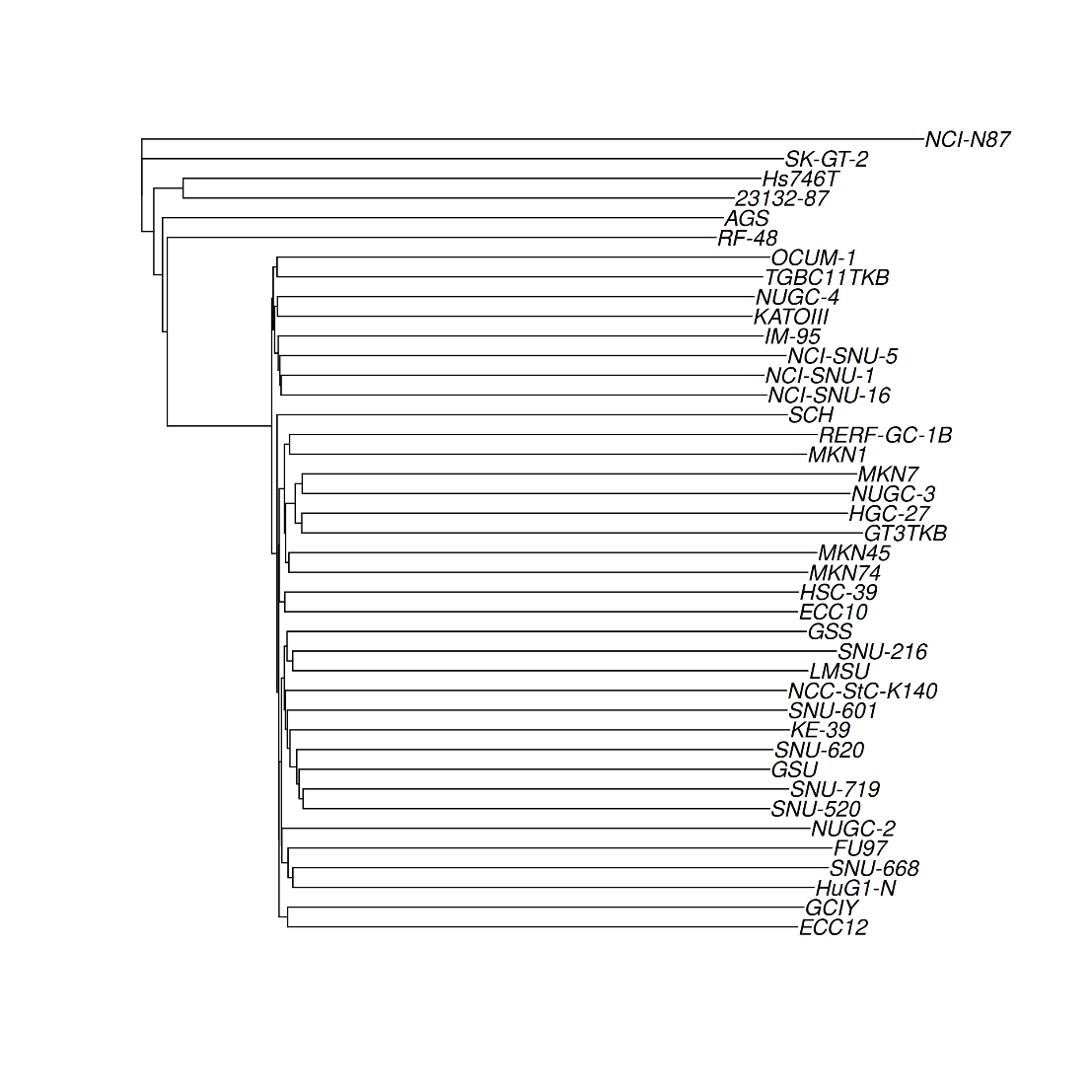


Supplementary Figure S2- SNP based phylogenetic tree. Phylogenetic tree depicting the SNP distance between the 41 CCLE gastric cancer cell lines.


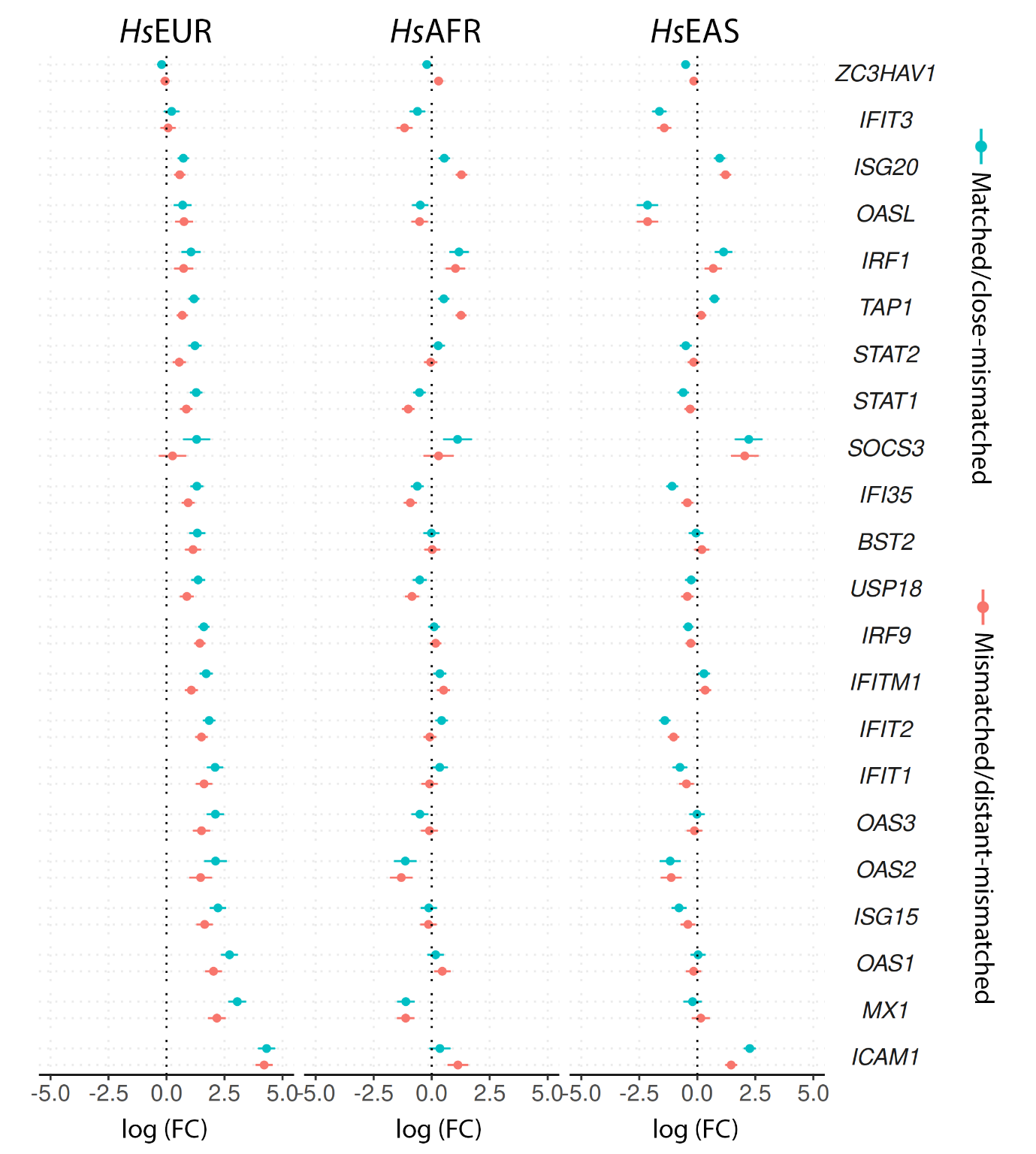


Supplementary Figure S3- Log_2_ changes in gene expression between *Hp*AFR and *Hp*EUR infection sets for the Type I interferon genes and Interferon stimulated genes


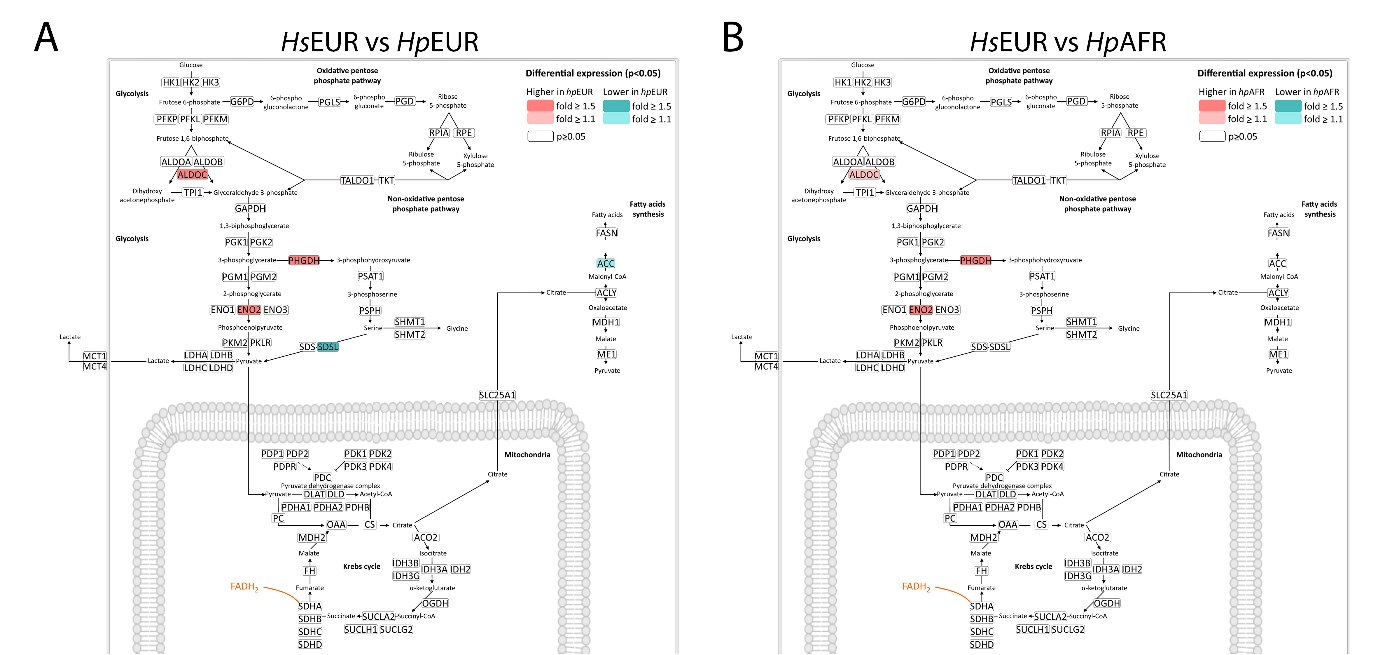


Supplementary Figure S4- Metabolism of lactate in the *Hs*EUR cell line. Statistically significant fold changes (up-regulation in pink and down-regulation in blue) in gene expression upon infection with **A**- *Hp*EUR and **B-** *Hp*AFR strains against the uninfected set in glycolysis, Krebs cycle, and fatty acids synthesis.


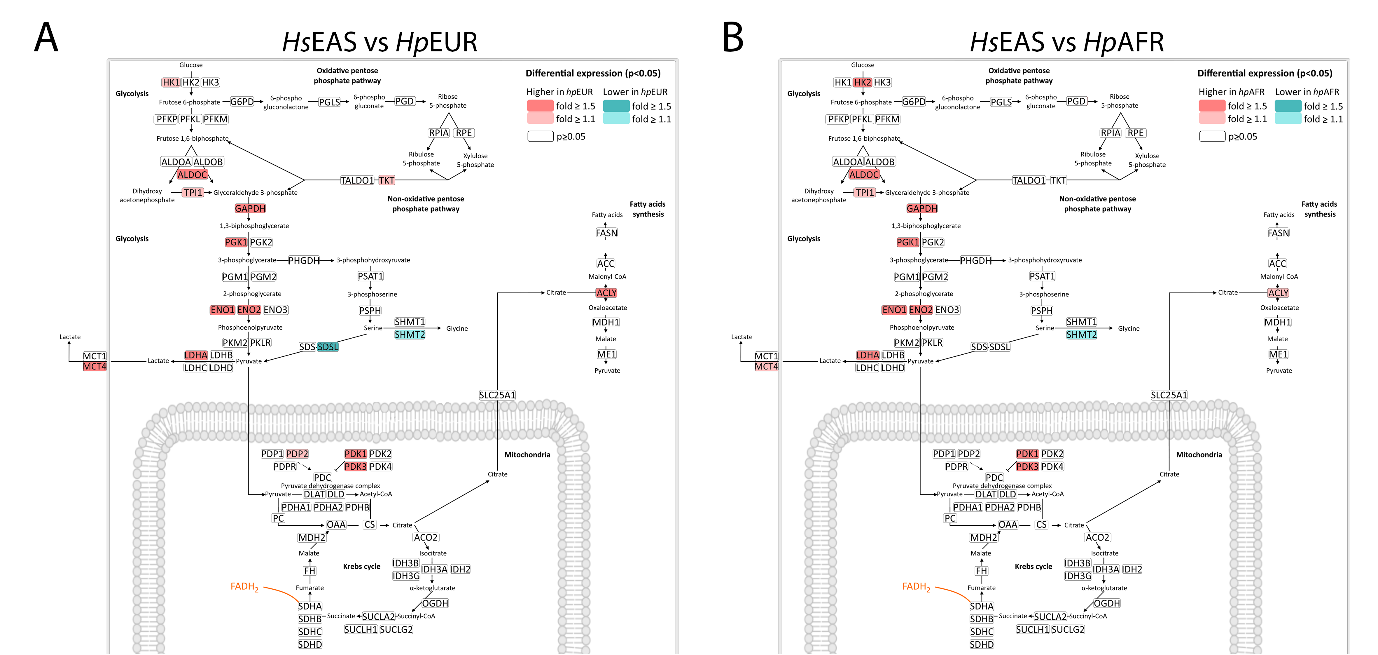


Supplementary Figure S5- Metabolism of lactate in the *Hs*EAS cell line. Statistically significant fold changes (up-regulation in pink and down-regulation in blue) in gene expression upon infection with **A**- *Hp*EUR and **B-** *Hp*AFR strains against the uninfected set in glycolysis, Krebs cycle, and fatty acids synthesis.


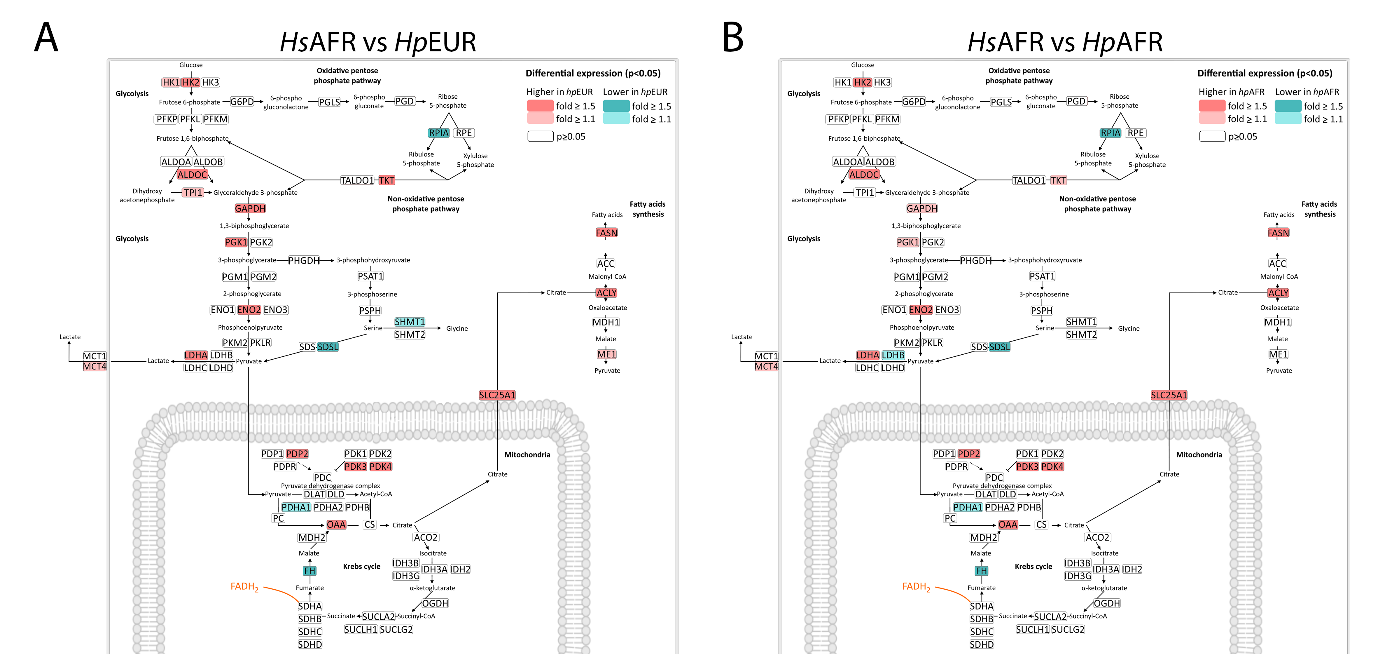


Supplementary Figure S6- Metabolism of lactate in the *Hs*AFR cell line. Statistically significant fold changes (up-regulation in pink and down-regulation in blue) in gene expression upon infection with **A**- *Hp*EUR and **B-** *Hp*AFR strains against the uninfected set in glycolysis, Krebs cycle, and fatty acids synthesis.

Supplementary Figure S7- Lactate cellular concentration (normalized mass-spectrometry values; see original paper for description) amongst 37 CCLE stomach cell lines, based on metabolomic data from Li et al. (2019). *Hs*EUR in blue, *Hs*EAS in green and *Hs*AFR in red.


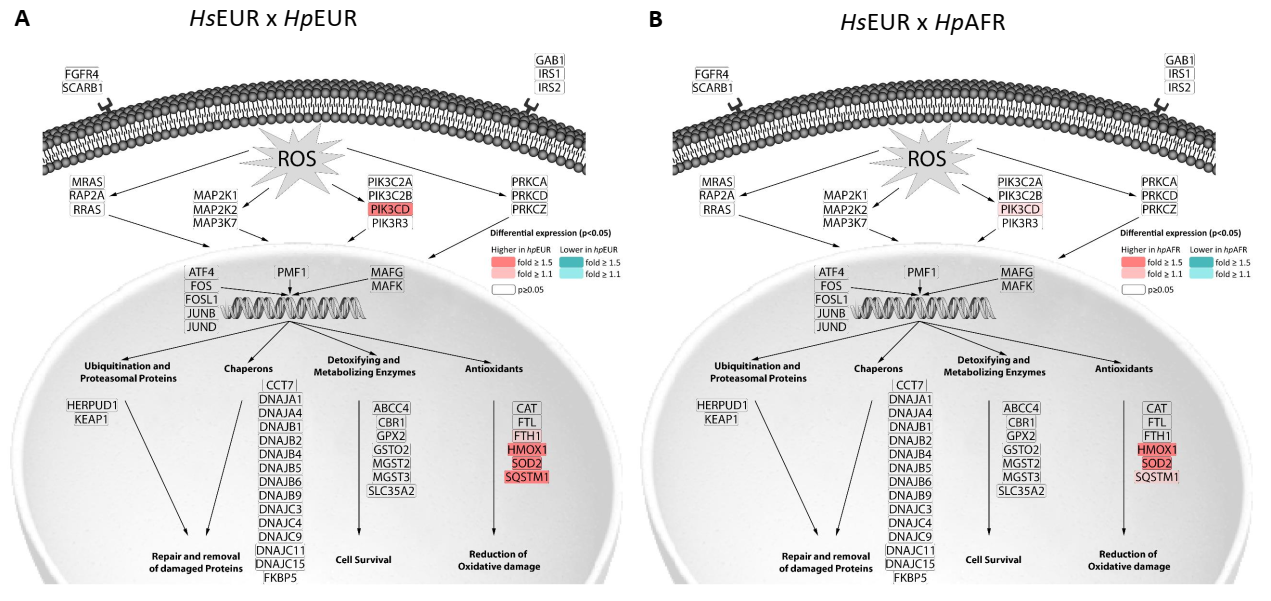


Supplementary Figure S8– Illustration of the oxidative stress response in the *Hs*EUR cell line. Statistically significant fold changes (up-regulation in pink and down-regulation in blue) in gene expression upon infection with **A**- *Hp*EUR and **B-** *Hp*AFR strains against the uninfected set.


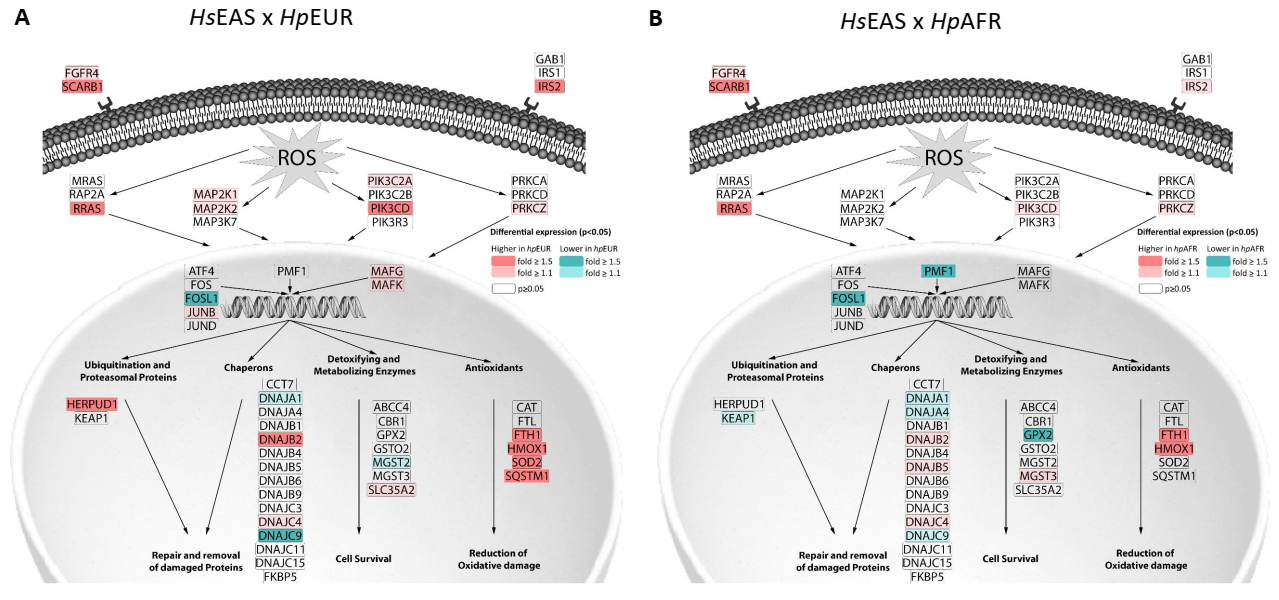


Supplementary Figure S9– Illustration of the oxidative stress response in the *Hs*EAS cell line. Statistically significant fold changes (up-regulation in pink and down-regulation in blue) in gene expression upon infection with **A**- *Hp*EUR and **B-** *Hp*AFR strains against the uninfected set.


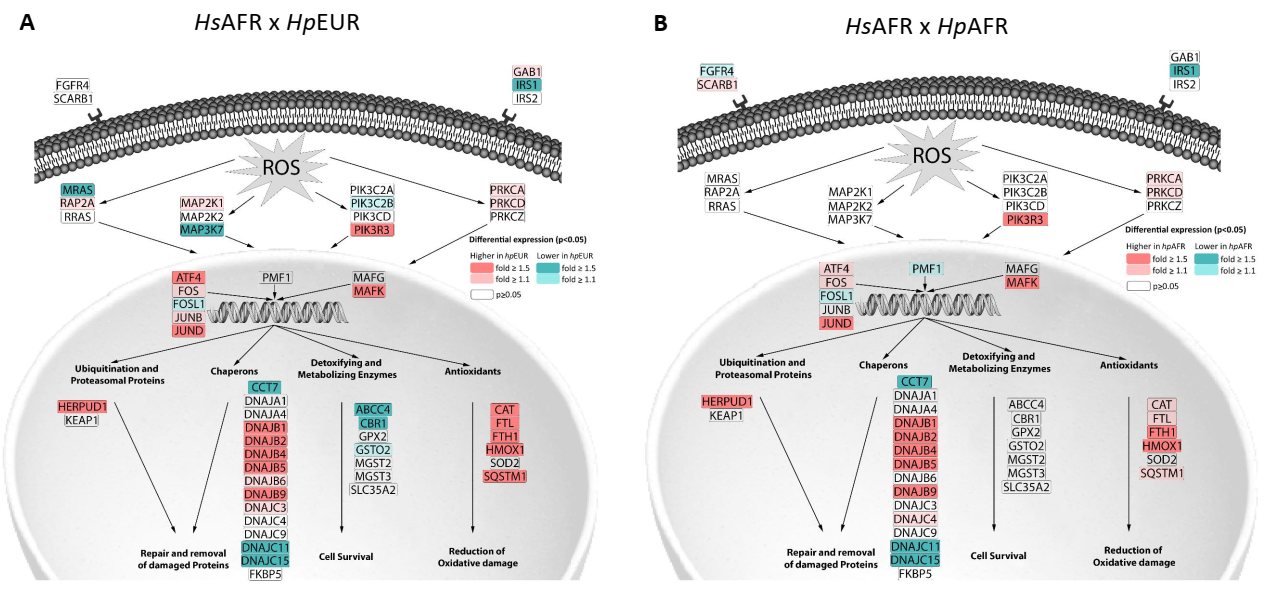


Supplementary Figure S10– Illustration of the oxidative stress response in the *Hs*AFR cell line. Statistically significant fold changes (up-regulation in pink and down-regulation in blue) in gene expression upon infection with **A**- *Hp*EUR and **B-** *Hp*AFR strains against the uninfected set.
